# The Behavior of Magnetic Properties in the Clusters of 4d Transition Metals

**DOI:** 10.3390/molecules23081896

**Published:** 2018-07-29

**Authors:** Habte Berry, Baolin Wang, Qinfang Zhang

**Affiliations:** 1 School of Materials Science and Engineering, Yancheng Institute of Technology, Yancheng 224051, China; habtix07@gmail.com; 2 Department of Physics, College of Natural and Computational Sciences, Dilla University, P.O. Box 419, Dilla, Ethiopia; 3 School of Physical Science and Technology, Nanjing Normal University, Nanjing 210023, China; wbl@ycit.edu.cn

**Keywords:** critical size, tight-binding Friedel model, stoner criterion, Heisenberg model

## Abstract

The current focus of material science researchers is on the magnetic behavior of transition metal clusters due to its great hope for future technological applications. It is common knowledge that the 4d transition elements are not magnetic at their bulk size. However, studies indicate that their magnetic properties are strongly dependent on their cluster sizes. This study attempts to identify magnetic properties of 4d transition metal clusters. Using a tight-binding Friedel model for the density of d-electron states, we investigated the critical size for the magnetic-nonmagnetic transition of 4d transition-metal clusters. Approaching to the critical point, the density of states of the cluster near the Fermi level is higher than 1/J and the discrete energy levels form a quasi-continuous band. Where J is correlation integral. In order to determine the critical size, we considered a square shape band and fcc, bcc, icosahedral and cuboctahedral close-packed structures of the clusters. We also investigated this size dependent magnetic behavior using Heisenberg model. Taking some quantum mechanical approximations in to consideration, we determined magnetic behavior of the clusters. For practicality, we considered three clusters of transition metals (Ru, Rh and Pd) and the obtained results are in line with the results of previous studies.

## 1. Introduction

It can be reckoned that magnetic properties of clusters of transition metal atoms are dependent on different internal factors such as the bond length, number of nearest neighbor interactions with the *d*-band electrons, the number of atoms in the cluster (*N*) and the symmetry of the cluster. Therefore, each of the above-mentioned factors are an independent parameter and contributes to the total energy $\left(E_{N}\right)$ of the cluster. The astonishing magnetic and electronic properties of transition metal clusters have greatly attracted the attention of researchers in the area in the last decade [1,2,3,4,5,6,7,8,9,10,11,12,13,14]. Obviously, these mysterious properties are results of a transition from bonds to bands as the atoms form clusters and then become the bulk solid. The small size and effective coordination number as well as high symmetry [14] are anticipated to give rise to narrow band and enhanced magnetism. For instance, studies in the 3d ferromagnetic transition metal cluster both experimental [1,2,3] and theoretical [4,5,6] investigations, yielded improved magnetic moments with respect to their bulk values. Although all d-block (3d, 4d and 5d) transition metals have unfilled localized d states; only certain 3d metals can form magnetic solids. However, none of the 4d or 5d elements are magnetic. If they could be made magnetic, they might provide a new class of magnetic materials with enhanced magneto crystalline anisotropy. Therefore, it is possible to magnetize groups of 4d atoms, as demonstrated by the discovery of giant induced moments in Pd [15]. Recently, Cox and co-workers have demonstrated that the Rh clusters exhibit spontaneous magnetic order [10,12]. This is the first case where a nonmagnetic solid has been shown to be magnetic as a cluster. Since the bulk material of Rh is non-magnetic, one may anticipate that a large Rh cluster must undergo a magnetic-non-magnetic transition at some critical size and then lose its moment.

Free atoms of the 3d transition metals have an incomplete d shell in the ground state or in excited states of small energy. Hence, the d electrons are responsible for the most interesting properties of these elements as free atoms or in the metallic bulk phase [16].

Recently, the study of the magnetic behavior of transition metal (TM) clusters has attracted the attention of material science researchers due to possible technological applications in electronic devices, magnetic recording, environmental processes and so on forth [17,18,19,20,21,22]. Though there are so many research outputs on magnetic behavior of these materials, nobody knows the exact mechanism for changing magnetic properties of the materials.

The search for magnetic behavior in transition metals has basically focused on the effects of reduced dimension. Atomic clusters represent an important class of reduced dimensionality and have produced some unexpected results bearing on magnetic ordering in small systems.

In general, this work will focus on the investigation of the magnetic properties of the aforementioned materials using Heisenberg ferromagnetism. We used basic quantum mechanical approximation in order to determine the thermodynamic and magnetic behaviors of the materials. In addition to this, the considered 4d transition metals are nonmagnetic at their bulk size and at the same time, findings from some research has indicated that there is magnetic–nonmagnetic transition in the materials. Using well known approximations and criterion we determined the critical size of the materials and investigated their magnetic behaviors.

## 2. Determination of Critical Size for Magnetic-Non-Magnetic Transition

In order to determine the magnetic to non-magnetic transition in non-magnetic 3d and 4d metal clusters, we used a tight-binding Friedel model of d-band and Stoner criterion [23,24,25]. The transition from a magnetic to a non-magnetic property takes place when the number of states per interval of energy [6,23,24,25] at the Fermi level is sufficiently high so that the highest occupied electronic level and the lowest unoccupied level overlap by virtue of the thermal width, J. Based on this assumption, even if the outermost shell electrons are delocalized over the entire cluster, a small cluster may remain magnetic until the density of states near the Fermi energy exceeds 1/J. In transition-metal clusters, the quasi-localized nature of d band is reckoned to be the cause for the electronic and magnetic properties of the cluster. Hence, we used the tight-binding Friedel model and assume the d-band width of an n atom in the cluster is l(n). Therefore, the total density of states of the square band can be expressed as [25,26]:

(1) $$\rho \left(\epsilon \right)=\{\begin{array}{c} \frac{10}{l\left(n\right)}\;for\;\frac{-l\left(n\right)}{2}<E-E_{d}<\frac{l\left(n\right)}{2},\\ 0\;otherwise. \end{array}$$

Here $E_{d}$ is the energy of the d-level and it is considered the zero point of energy and l(n) is the local bandwidth (assumed Equation ual for up and down spins). The number 10 refers to the total number of electrons in the atomic d-shell. The dependence of the band width on the local environment is introduced by using the second moment approximation and according to second moment approximation in the tight-binding theory [25,26] l(n) is proportional to the square root of the effective local coordination number Z(n):

(2) $$l\left(n\right)=l_{b}{\left(Z\left(n\right)/Z_{b}\right)}^{1/2},$$

where $l_{b}$ and $Z_{b}$ are band width of d-level and the coordination number of the bulk solid, respectively. Substituting Equation (2) in Equation (1), the density of states at Fermi level $\left(E_{F}\right)$ for the cluster can be expressed as a function of the effective coordination number,

(3) $$\rho \left(E_{F}\right)=\frac{10}{l_{b}}{\left[\frac{Z_{b}}{Z\left(n\right)}\right]}^{1/2}$$

In order to identify the presence of ferromagnetic behavior in such system we can implement the simple Stoner criterion [27] and it can be stated as, for a system to exhibit ferromagnetism, the density of states at the Fermi energy needs to be satisfied:

(4) $$J\rho \left(E_{F}\right)-1\geq 0,$$

where J is the correlation energy [28]. If we assume the magnetic-non-magnetic transition takes place at some critical cluster size $n_{c},$ then $\rho \left(E_{F}\right)$ of the cluster should satisfy the criterion

(5) $$\frac{10J}{l_{b}}{\left[\frac{Z_{b}}{Z\left(n_{c}\right)}\right]}^{1/2}=1.$$

Therefore, the effective coordination number at the critical size will be:

(6) $$Z\left(n_{c}\right)=Z_{b}{\left[\frac{10J}{l_{b}}\right]}^{2}.$$

The effective nearest-neighbor coordination number Z of atoms in a cluster is an important parameter characteristic of cluster size and shape. Therefore, the mean coordination number as a function of small cluster size (*n*) can be expressed as [29,30,31]:

(7) $$Z\left(n\right)=\frac{n-1}{1+\frac{n-1}{12}}$$

Here, we consider face centered cubic (fcc) and body centered cubic (bcc) structures [28]. There is a very large effect of geometry on the calculation of critical size in magnetic to non-magnetic transition. Even if there is no clear information about the geometric structures of transition-metal clusters, some research has indicated [29] that stable structures of such clusters tend to adopt icosahedral or cuboctahedral close-packed structures in the medium size range. Obviously, below the critical size, the density of states for the cluster as a function of Fermi energy $\rho \left(E_{F}\right)$ is greater than 1/J and the transition towards the ferromagnetic behavior takes place. Hence the critical size can be written as:

(8) $$n_{c}=\frac{{\left[\frac{10J}{l_{b}}\right]}^{2}\frac{11Z_{b}}{12}+1}{1-\frac{Z_{b}}{12}{\left[\frac{10J}{l_{b}}\right]}^{2}}$$

Now the critical radius of the cluster will be $R_{c}=R_{w}n_{c}{}^{1/3}.$ Where $R_{w}$ the Wigner-Seitz radius. The effective coordination number of atoms in a cluster is an important parameter characteristic of cluster size and shape. Therefore, it is important to consider the geometry of a cluster during effective coordination number calculation. Now here let’s assume that icosahedron and Cuboctahedron geometries of the clusters. The effective coordination numbers [32] for the two geometries are:

(9) $$Z\left(n\right)=\frac{6\left(n-1\right)\left(20n^{2}-25n+12\right)}{\left(2n-1\right)\left(5n^{2}-5n+3\right)},$$

and

(10) $$Z\left(n\right)=\frac{12\left(n-1\right)\left(10n^{2}-14n+6\right)}{\left(2n-1\right)\left(5n^{2}-5n+3\right)},$$

for icosahedron and cub-octahedron respectively. Taking in to account of these expressions, we can determine the critical size and radius of the clusters.

Now let us consider that the d-band splitting energy ($∆E=\left|\epsilon_{d}^{↑}-\epsilon_{d}^{↓}\right|$) caused by the exchange interaction is independent of cluster size and Equation ual to the bulk value, then the local magnetic moment:

(11) $$\mu_{i}=\int_{-\infty }^{\epsilon_{F}}\left[\rho_{n}^{↑}\left(\epsilon \right)-\rho_{n}^{↓}\left(\epsilon \right)\right]d\epsilon ,$$

and it become

(12) $$\mu_{i}=\{\begin{array}{c} {\left(\frac{Z_{b}}{Z\left(n\right)}\right)}^{1/2}\mu_{b},\;if\;Z\left(n\right)\geq Z_{c}\\ \mu_{\dim\mathrm{otherwise}}. \end{array}$$

where $Z_{c}$ is the critical coordination number below which the magnetic moment of that atom adopts the value $\mu_{dim}$ of the dimer. Therefore, the average magnetic moment of the cluster will be:

(13) $$\bar{\mu_{n}}=\frac{1}{n}\sum_{i=1}^{n}\mu_{i}.$$

In a system of magnetic moments of atoms in a cluster, the relative magnetization can be defined as $\gamma_{m}=M\left(T\right)/M\left(0\right)$; usually it serves as a measure of ordering (the order parameter). In such a case, the disordering is also represented by the relative variation of the magnetization:

(14) $$M\left(0\right)-M\left(T\right)/M\left(0\right)=∆M\left(T\right)/M\left(0\right)=1-\gamma_{m}.$$

This means that according to the Bloch law at low temperatures, it is proportional to $T^{3/2}$ [33]. Increase in temperature implies that an enhancement of the disordering in the magnetic system and leads to an increase in the entropy $\left(\varsigma_{m}\left(T\right)\right)$. To the completely disordered (paramagnetic) statefrom ferromagnetic state, the entropy change of magnetic system can be expressed as:

(15) $$∆\varsigma_{max}=Rln\left(2s+1\right),$$

where R is the universal gas constant and *s* is the spin of the electron [34].

But here, the ratio $\varsigma_{m}\left(T\right)/∆\varsigma_{max}$ can be considered as a measure of disordering of the magnetic system. Hence, the ordering in the system will be characterized by the quantity:

(16) $$\gamma_{\varsigma }=1-\varsigma_{m}\left(T\right)/∆\varsigma_{max}.$$

Therefore, by assuming that, in the low temperature range the values of $\gamma_{m}$ and $\gamma_{\varsigma }$ are Equation ual to each other and we obtain:

(17) $$M\left(T\right)/M\left(0\right)=1-\varsigma_{m}\left(T\right)/∆\varsigma_{max}.$$

Using Equations (14)–(17) we derive expression for magnetic entropy as:

(18) $$\varsigma_{m}\left(T\right)=Rln\left(2s+1\right)\left(M\left(T\right)/M\left(0\right)\right).$$

By using Equation (18) and the well-known thermodynamic relationship:

(19) c=T(dς/dT),

where *c* is the heat capacity. Consequently, we obtain the following mathematical relation for the magnetic component of the heat capacity al low temperatures:

(20) $$c_{m}\left(T\right)=RTln\left(2s+1\right)\frac{d}{dT}\left(∆M\left(T\right)/M\left(0\right)\right).$$

If the expression of the Bloch law for the relative change in the magnetization is given by:

(21) $$∆M\left(T\right)/M\left(0\right)=\left(0.0587/sQ\right){\left(k_{B}T/2Js\right)}^{3/2}=AT^{3/2},$$

and the exchange correlation integral J can be obtained from the mean field approximation as:

(22) $$J=3k_{B}T_{c}/2\bar{Z}\;s\left(s+1\right).$$

In Equation (21) *A* is a constant dependent on the type of cluster structure (Q), the spin of the electrons and the character of interaction in the magnetic system of the material and s=1/2, Q=4 (for fcc structure). After substitution into relationship (20), we get the well known three halves power law for the low temperature magnetic component of the heat capacity of the ferromagnet:

(23) $$c_{m}\left(T\right)=RTln\left(2s+1\right)(d/dT)AT^{3/2}=BT^{3/2}.$$

## 3. Theoretical Model

As a governing dynamic, we can consider the Heisenberg model for small clusters of transition metals [35,36]:

(24) $$H=-J\sum_{i>j}S_{i}\cdot S_{j}-h\sum_{i}S_{i}^{z}.$$

where in this Hamiltonian we can assume that, the first summation is only for nearest neighbor interactions of the cluster but let us consider the second summation is over all atoms. In such a kind of isotropic model, an effective interaction coupling (J) between spins according to molecular-field theory is approximated to be proportional to the bulk Curie temperature $kT_{c}^{bulk}.$ The second component of the Hamiltonian is due to external magnetic field (the Zeeman term) where $S_{i}^{z}$ is the component of spin. However, this Hamiltonian is quite general and it should be particularized to either the classical (Stending to infinity) or the quantum case. In order to perform this the first step must be evaluating the partition function: $\zeta =Tre^{-H/kT}$. If we can find expression for the partition function, then it is possible to determine the thermodynamic properties, magnetization and specific heat capacity of the system. Here our main target is to acquire a better understanding into the physics of small clusters of transition metals it is quite useful to start with some important assumptions valid for small systems, in which case the Heisenberg Hamiltonian can be solved analytically for quantum spins. A cluster is nothing but small, multi-atom particles. Therefore, if we neglect the usually subordinate, purely magnetic, coupling between spin and orbital angular momenta, the total spin of the entire cluster is conserved and the square of the spin has the characteristic values [36] $S^{\prime }\left(S^{\prime }+1\right)$ where $S^{\prime }$ is a whole or half-integer according to the number of electrons in the entire cluster being even or odd.

Now let’s assume that the square of the total spin operator ${\left(S^{\prime }\right)}^{2}$ of the cluster is given by:

(25) $${\left(\sum_{i}S_{i}\right)}^{2}=\sum_{i}S_{i}^{2}+\sum_{i\neq j}S_{i}\cdot S_{j}=S^{\prime }\left(S^{\prime }+1\right).$$

Equation (25) will provide an easy solution to the considered Hamiltonian in the case each atom is coupled to every other atom in the cluster. Hence this supposition can be applied only for relatively small clusters. Based on this, the Eigen values of the Hamiltonian will be:

(26) $$E\left(S,S^{\prime },M^{\prime }\right)=\frac{1}{2}J\left[NS\left(S+1\right)-S^{\prime }\left(S^{\prime }+1\right)\right]+hM^{\prime },$$

where $h=g\mu_{B}H.\;M^{\prime }$ is the z-axis component the total angular momentum and its Eigen values are $M^{\prime }=S^{\prime },S^{\prime }-1,\;\ldots ,\;-\left(S^{\prime }-1\right),\;-S^{\prime }$ (spin of the cluster) where $S^{\prime }=0,1,2,\ldots ,NS$ (spin of the atom) for *NS* integral and $S^{\prime }=1/2,3/2,5/2,\ldots ,\;NS$ for *NS* half integral. Hence using these parameters, the partition function for the clusters is:

(27) $$\zeta =\sum_{S^{\prime }}\sum_{M^{\prime }}\sigma (N,S,S^{\prime })e^{J\beta S^{\prime }\left(S^{\prime }+1\right)/2}e^{h\beta M^{\prime }},$$

$\beta^{-1}$ = kT. In Equation (27) we can ignore terms as function of only S since such terms are of no interest for our problems in magnetism. $\sigma \left(N,\;S,\;S^{\prime }\right)$ is the number of accessible states to combine [35] *N* spin *S* to obtain a total spin $S^{\prime }$

(28) $$\zeta \left(J,h\right)=\sum_{S^{\prime }}\sigma \;e^{J\beta S^{\prime }\left(S^{\prime }+1\right)/2}\frac{Sinh[(S^{\prime }+1/2)h\beta ]}{Sinh\left(\frac{h\beta }{2}\right)}=\sum_{S^{\prime }}P(J,h,S^{\prime }),$$

where $P\left(J,h,S^{\prime }\right)=\sigma \;e^{J\beta S^{\prime }\left(S^{\prime }+1\right)/2}\frac{Sinh[(S^{\prime }+1/2)h\beta ]}{Sinh\left(\frac{h\beta }{2}\right)}$.

Using statistical mechanics definition of magnetization:

(29) $$M=\beta^{-1}\frac{\partial \left(ln\zeta \right)}{\partial H};T=constant.$$

By substituting the expression for partition function of Equation (28) in Equation (29) then, the magnetization (mean magnetic moment per unit volume) will be:

(30) $$M=g\mu_{B}\frac{\sum_{S^{\prime }}P\left(J,h,S^{\prime }\right)S^{\prime }B_{S^{\prime }}\left(h,\beta ,s^{\prime }\right)}{\sum_{S^{\prime }}P(J,h,S^{\prime })}.$$

where $B_{S^{\prime }}$ is the Brillouin function and it is expressed as:

(31) $$B_{S^{\prime }}\left(h,\beta ,s^{\prime }\right)=\frac{2s^{\prime }+1}{2s^{\prime }}\coth\left(\frac{2s^{\prime }+1}{2s^{\prime }}\left(\frac{gh\mu_{B}s^{\prime }}{k_{B}T}\right)\right)-\frac{1}{2s^{\prime }}\coth\left(\frac{1}{2s^{\prime }}\left(\frac{gh\mu_{B}s^{\prime }}{k_{B}T}\right)\right).$$

Therefore, expression for magnetization in (Equation (30)) is valid for any value of spin *S*. However, for large cluster sizes, individual atoms may no longer be the nearest neighbors of each other—the characteristic Equation (Equation (25)) cannot be used anymore and the geometry of the cluster must be considered explicitly. Hence, the general form of the magnetization will be [37,38]:

(32) $$M=\frac{\sum^{}g\left(y\right)f\left(y\right)}{\sum^{}g\left(y\right)},$$

and Equation (30) is still conserved.

If we assume that $S^{\prime }\to \infty$ (implies that, when all orientations of the magnetic moment become possible), the phenomenon of super paramagnetism appears as a weighted average of Langavin [37] $L\left(h\beta S^{\prime }\right)$ contributions of spin $S^{\prime }$ with:

(33) $$L\left(x\right)=cothx-\frac{1}{x}.$$

As the original work of Heisenberg approximated [35], assuming that for big systems (large *N*), the partition function has a maximum at some particular value of $S^{\prime }$ denoted $\hat{S}$; hence, it is only sufficient to take terms with the first order in the Taylor expansion about $\hat{S}$ and we obtain for magnetization:

(34) $$M=g\mu_{B}\hat{S}L\left(h\beta \hat{S}\right).$$

But $L\left(h\beta \hat{S}\right)=\frac{1}{3}\frac{gh\mu_{B}\hat{S}}{k_{B}T}-\frac{1}{45}{\left(\frac{gh\mu_{B}\hat{S}}{k_{B}T}\right)}^{3}+\ldots$

The normalized magnetic moment per site, calculated from Equation (30), is represented as a function of temperature for various sizes *N* will be:

(35) $$M\approx g\mu_{B}\hat{S}\frac{1}{3}h\beta \hat{S}\;\;or\;M\approx \frac{gh{\hat{S}}^{2}\mu_{B}}{3k_{B}T}.$$

But $h=g\mu_{B}H.$ Hence the approximate value of the magnetization will be:

(36) $$M\approx \frac{{\left(g\mu_{B}\hat{S}\right)}^{2}H}{3k_{B}T}.$$

One of the fundamental problems of the Heisenberg theory of ferromagnetism is how to determine the characteristic values of the considered Hamiltonian and hence the energy states belonging to various resultant spins of the cluster. Before explaining the mathematical details of how this is done, it will perhaps be illuminating to consider the following limiting case: at high temperature ($J/k_{B}T\ll 1$). In this limiting case, thermal kick is higher than that of the exchange coupling energy by far, so that J is negligible and the susceptibility will be:

(37) $$\chi =4NS\left(S+1\right)\eta^{2}/k_{B}T.$$

where $\eta =g\mu_{B}\hat{S}$.

In order to find the critical temperature (the Curie point) and for the paramagnetism at high temperatures, one can expand expression for the Brillouin function (Equation (31)) as a power series:

(38) $$B_{S^{\prime }}\left(h,\beta ,s^{\prime }\right)=\frac{1}{3}\frac{\left(S+1\right)\delta }{S}-\frac{1}{720S^{4}}\left[{\left(2S+1\right)}^{4}-1\right]\delta^{3},\;\delta =\frac{2\beta SH}{k_{B}T}+\frac{2JZS^{2}B_{S^{\prime }}\left(h,\beta ,s^{\prime }\right)}{k_{B}T}.$$

As explained above, the first order term of the expansion of the two equations of (38) must become identical for H=0 at the Curie point $T_{c}$. Hence the critical temperature will be:

(39) $$T_{c}=\frac{2JZ}{3k}S\left(S+1\right)$$

## 4. Behavior above Curie Point

According to Equation (38), we can expand the Brillouin function using Taylor series. At high temperatures, it is adequate to take only the linear portion of Equation (38). Hence, we can eliminate δ between the two parts of Equation (38) very easily and it is thus found on using Equation (39) that the expression for the susceptibility:

(40) $$\chi =\frac{4N\eta^{2}S\left(S+1\right)}{3k\left(T-T_{c}\right)}.$$

Therefore, Equation (40) is well known Curie-Weiss law of magnetic susceptibility. Using Heisenberg model and the approximation in Equations (25) and (26) we can obtain the magnetic susceptibility and the Curie temperature. As we can see from Equations (39) and (40) the results are exactly the same as previous studies.

## 5. Discussion

In this paper, we considered three clusters of 4d transition metals (Ru, Rh and Pd) and studied their magnetic behavior at critical size and below. Obviously, the low dimensionality and reduced coordination number in a transition metal cluster are expected to give rise to narrow d-band (and consequently sharper density of states) with regard to the bulk band. Hence, we used the tight-binding Friedel model and Stoner criterion for ferromagnetism. By assuming a square d-band and face centered cubic (fcc) like (or icosahedral, cuboctahedral) close-packed atomic structures in transition metal clusters, we obtained an empirical formula for critical size (Table 1 and Table 2) and the results are consistent with previous experimental and theoretical studies [25]. As we can see from Table 2, during critical size determination we have taken the cluster structure in to consideration. Hence there is a critical size variation due to structure change of some materials. In general, the values of critical size in both tables are different, especially for Rh cluster. This is due to the approximated coordination number [32] used in Equation (7). The other reason is because we did not take in to account the exact cluster geometry in the case of Equation (7). All in all, we identified some important weaknesses in Equation (7). Firstly, the expression (Equation (7)) only relays on the number of atoms in the cluster and did not take in to account the cluster geometry. Secondly, due to the irregular pattern of atomic coordination numbers on the cluster surface, it can be misleading to use the expression for number of atoms representing clusters with in complete shells of atoms. Thirdly, the expression is not precise as it increases towards the limiting bulk value of 12 much too quickly.

**Figure 1:**
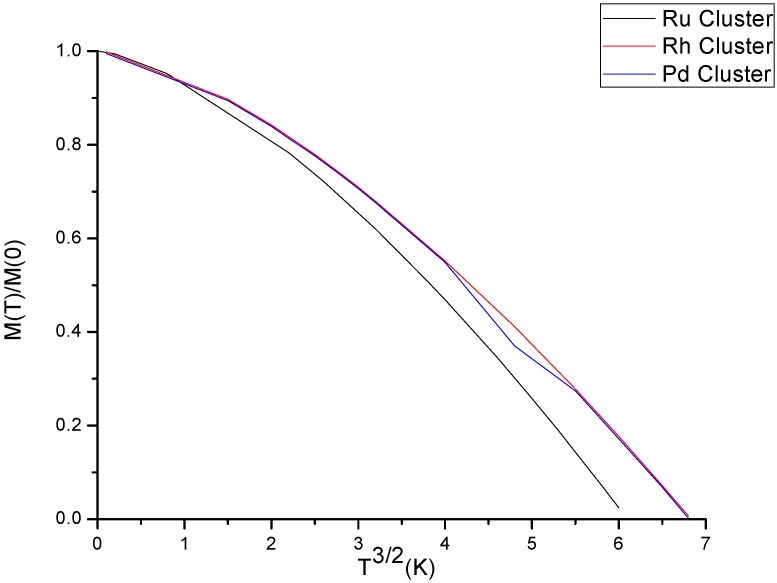
Shows the dependence of relative magnetization on the temperature for the three clusters.

**Figure 2:**
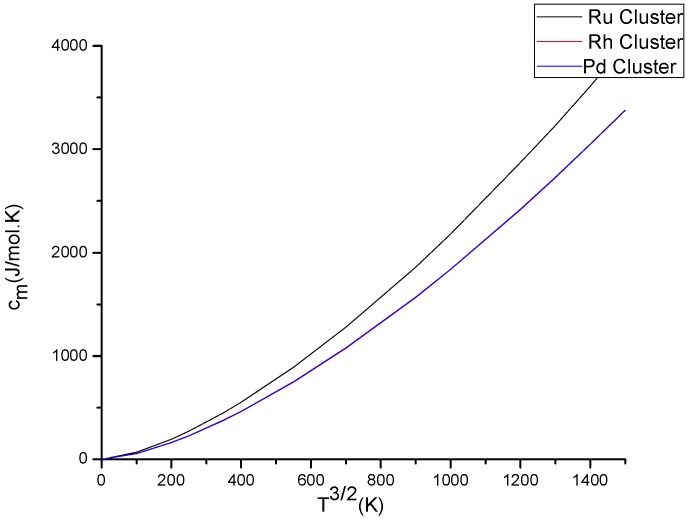
Explains the variation of heat capacity with temperature for the three clusters.

When the temperature is getting low, that is, at the absolute zero of temperature, the magnetic moments of a ferromagnet are in a completely ordered state. This means the system has maximum value of the spontaneous magnetization M(0) Figure 1 and the zero entropy of the magnetic system. With an increase in the temperature, the thermal kick disturbs the ordering in the magnetic system and the magnetization M(T) decreases. Hence for the considered clusters, the mean magnetic moment (magnetization) shows the same characteristics with the subject of existing science in the area for small clusters sizes. We also studied the property of magnetic heat capacity (Figure 2) of the clusters. According to Bloch law, magnetic heat capacity of ferromagnetic materials is proportional to three-halves power law ($\sim T^{3/2}$). As we can see from Figure 2, at low temperatures, the values of the magnetic contribution to the heat capacity of the materials are in agreement with previous findings. that is, the heat capacity vanishes at absolute zero and at low temperatures it is proportional to the absolute temperature. As the temperature increases, the values of magnetic heat capacity become increasingly higher than that of the values at low temperatures.

**Figure 3:**
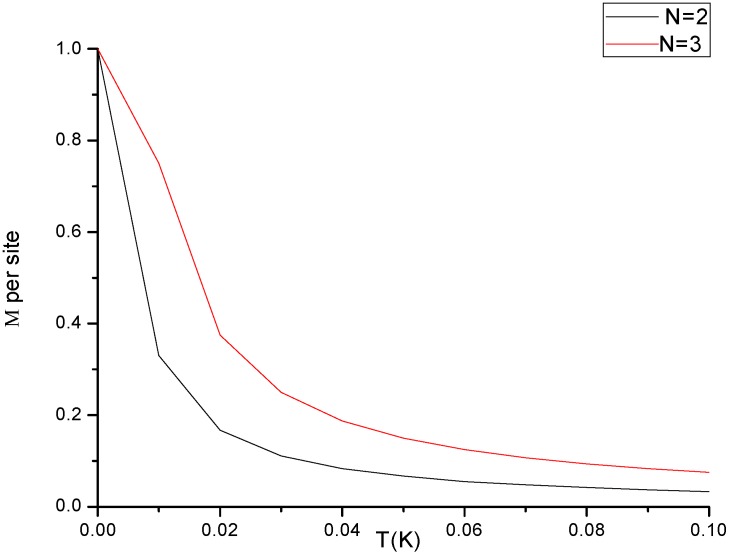
Shows the variation of magnetization(M) with temperature for small cluster sizes.

In order to cross check the results (magnetic properties), we have used Heisenberg model and the results (Figure 3) are in line with previous studies for small cluster seizes (specifically for cluster sizes *N* ≤5 the model is applicable to study such phenomenon). The assumption in the Heisenberg model is only valid for relatively small clusters this is because individual atoms may no longer be nearest neighbors of each other for large cluster sizes. Since the spontaneous symmetry breaking at $T_{c}$ can occur in the thermodynamic limit only (N→∞) the net alignment does not drop to zero at $T=T_{c}$ as indicated in Figure 3. Spin fluctuations lead to some small alignment even at high temperatures. At very low temperatures, quantum mechanical effects normally come into play. However, the standard Bloch $T^{3/2}$ law is only obeyed approximately in clusters.

**Figure 4:**
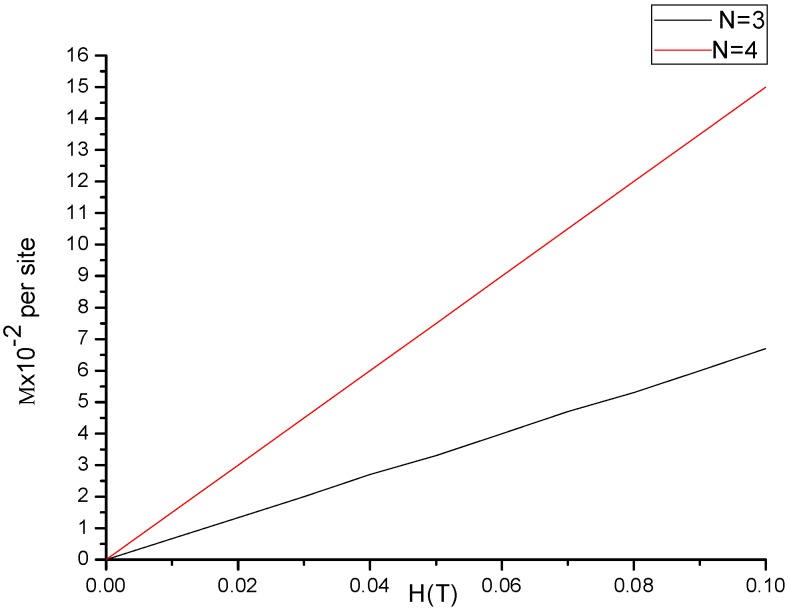
Shows the dependence of magnetization(M) on magnetic field and the size of the cluster.

Figure 4 explains the averaged projection of spin per site on the field axis for small sized cluster at T=0.50 K, as a function of external magnetic field. The projection increases linearly with field until it saturates at the maximum value (i.e., when the cluster size is approaching to the critical size). Therefore, this result is in line with previous studies in magnetism.

## 6. Conclusions

We studied the magnetic to non-magnetic transition of 4d transition metal by using tight binding Friedel d-band model and Heisenberg model. We determined the critical size, critical radius and effective coordination number of the three 4d transition metals by using a square shape of d-band, cluster size dependent density of states and taking the geometry of the clusters in to account. We also studied the effective magnetization and magnetic heat capacity of the clusters and cross checked the results by applying Heisenberg model for the small clusters. The results showed that even if 4d transition metals are non-magnetic at their bulk size, they make a transition to magnetic at their small cluster sizes. In general, all the results are consistent with previous theoretical and experimental studies.

## Figures and Tables

**Table 1:** Some of the important parameters from our calculations and previous studies.

Clusters	Ru	Rh	Pd	Nb	Mo
Structure	fcc	fcc	fcc	bcc	bcc
$R_{c}$ (angstrom)	9.89	11.5	4.72	2.24	2.46
$l_{b}\left(eV\right)$	8.44 [25]	6.89 [25]	5.4 [25]	9.72 [25]	9.98 [25]
values of $n_{c}$ from previous studies	13 [8]	90 [10]	13 [8]	5 [25]	5 [25]
J(eV)	0.561	0.643	0.641	0.557	0.541
$n_{c}$	12	92	8	5	4
$Z\left(n_{c}\right)$	6	11	4	4	3
$\bar{\mu_{n_{c}}}\;\left(\mu_{b}\right)$	≤0.121	≤0.012	≤0.21	≤0.31	≤0.44

**Table 2:** Effect of structure on the critical size of the clusters.

Structure of the Cluster	Shape of the Band	Critical Size of Ru	Critical Size of Rh	Critical Size of Pd
Face centered cubic	square	12	92	8
Icosahedron	square	9	1048	5
Cuboctahedron	square	14	1807	7
